# CD4^+^CD25^+^CD127^hi^ cell frequency predicts disease progression in type 1 diabetes

**DOI:** 10.1172/jci.insight.136114

**Published:** 2021-01-25

**Authors:** Aditi Narsale, Breanna Lam, Rosa Moya, TingTing Lu, Alessandra Mandelli, Irene Gotuzzo, Benedetta Pessina, Gianmaria Giamporcaro, Rhonda Geoffrey, Kerry Buchanan, Mark Harris, Anne-Sophie Bergot, Ranjeny Thomas, Martin J. Hessner, Manuela Battaglia, Elisavet Serti, Joanna D. Davies

**Affiliations:** 1 San Diego Biomedical Research Institute, San Diego, California, USA.; 2 Immune Tolerance Network, Bethesda, Maryland, USA.; 3 San Raffaele Diabetes Research Institute, Istituto di Ricovero e Cura a Carattere Scientifico (IRCCS) San Raffaele Hospital, Milan, Italy.; 4 Department of Pediatrics, Medical College of Wisconsin, Milwaukee, Wisconsin, USA.; 5 Diamantina Institute, University of Queensland, Woolloongabba, Queensland, Australia.; 6 Department of Pediatric Endocrinology, Queensland Children’s Hospital, South Brisbane, Queensland, Australia.

**Keywords:** Autoimmunity, Immunology, Autoimmune diseases, Diabetes, T cells

## Abstract

Transient partial remission, a period of low insulin requirement experienced by most patients soon after diagnosis, has been associated with mechanisms of immune regulation. A better understanding of such natural mechanisms of immune regulation might identify new targets for immunotherapies that reverse type 1 diabetes (T1D). In this study, using Cox model multivariate analysis, we validated our previous findings that patients with the highest frequency of CD4^+^CD25^+^CD127^hi^ (127-hi) cells at diagnosis experience the longest partial remission, and we showed that the 127-hi cell population is a mix of Th1- and Th2-type cells, with a significant bias toward antiinflammatory Th2-type cells. In addition, we extended these findings to show that patients with the highest frequency of 127-hi cells at diagnosis were significantly more likely to maintain β cell function. Moreover, in patients treated with alefacept in the T1DAL clinical trial, the probability of responding favorably to the antiinflammatory drug was significantly higher in those with a higher frequency of 127-hi cells at diagnosis than those with a lower 127-hi cell frequency. These data are consistent with the hypothesis that 127-hi cells maintain an antiinflammatory environment that is permissive for partial remission, β cell survival, and response to antiinflammatory immunotherapy.

## Introduction

Type 1 diabetes (T1D) is a progressive heterogeneous autoimmune disease resulting in the destruction of insulin-secreting β cells by T cells (1). Despite enormous effort, there is still no therapy to stop and reverse the disease. However, there are sufficient examples of immunotherapies that transiently preserve β cell function in subpopulations of patients to encourage the notion that disease progression can be modulated by targeting the immune system (2–6). In an effort to identify novel immune mechanisms that might be targeted therapeutically to treat and reverse T1D, our group has focused attention on a well-recognized natural phenomenon called partial remission, a period of improved glucose control experienced by many patients with T1D soon after diagnosis (7–10). Partial remission can last from several weeks to over 1 year (11, 12). The clinical significance for the partial remission period is that patients who experience partial remission have a significantly reduced risk of complications (13), including chronic microvascular complications (14), a lower risk of severe hypoglycemia (15, 16) and diabetic retinopathy (17), and an increase in growth in prepubescent children (18). Therefore, identifying mechanisms that promote and extend partial remission might also identify new targets for novel therapies to reduce short- and long-term complications.

The mechanism for partial remission is not known. One idea is that it is actively controlled by a form of immune regulation. Consistent with this idea, we have identified a CD4^+^ T cell population that expresses CD25 and a high density of CD127 that is present at a higher frequency in children with a long remission compared with children with short or no remission (19, 20). In healthy people, CD4^+^CD25^+^CD127^hi^ (127-hi) cells are predominantly memory cells with a mix of antiinflammatory (IL-4–, IL-10–, IL-13–, and IL-5–secreting) Th2 cells and proinflammatory (IFN-γ– and IL-2–secreting) Th1-type cells, with a bias toward Th2 cells (21). Th1 and Th2 cell function is defined by the cytokines that these cells secrete (22, 23). Thus, Th2 cytokines promote the differentiation to, and the expansion of, Th2 cells and inhibit the differentiation to and expansion of Th1 cells and vice versa (24–28). The Th2 bias in 127-hi cells might then suggest that in patients with T1D 127-hi cells play an active role in prolonging remission by promoting an antiinflammatory microenvironment.

The correlation between 127-hi cell frequency and remission length combined with the Th2 bias in 127-hi cell function provided the justification to test the validity of these findings in a larger new study. In collaboration with the Immune Tolerance Network (ITN); Medical College of Wisconsin (MCW); San Raffaele Diabetes Research Institute (SRDRI); and the University of Queensland (UQ), we tested the validity of our original finding that the relative frequency of 127-hi cells correlates with length of partial remission (LoR) (19).

CD4^+^ and CD8^+^ T cells fall into 3 main functional groups. Proinflammatory cells can be either IFN-γ–secreting Th1 (CD4^+^ T cells) and Tc1 (CD8^+^ T cells) cells or IL-17– and IL-22–secreting Th17/Tc17 cells; antiinflammatory Th2/Tc2 cells secrete IL-4, IL-5, and IL-13 and regulatory cells secrete IL-10 and TGF-β (29). Lineage-specific cytokines and transcription factors can positively influence their own expansion and negatively influence the expansion of other lineages. Specifically, in mice, it is well established that Th1 cells are positively induced by IL-12 and IFN-γ but negatively influenced by IL-4, while Th2 cells are positively regulated by IL-4 and negatively regulated by the Th1 cytokine IL-12 (22–27). In addition, the Th2-type transcription factor GATA-3 (30) plays a critical role in promoting the Th2 response and in inhibiting Th1 differentiation (31–34). Likewise, both IL-4 and IFN-γ inhibit the development of Th17 cells (35, 36), and, during an immune response, this results in a negative association between Th17 and both Th1 and Th2 responses. This type of immune regulation, or deviation, caused by the polarization toward and away from lineage commitment, can enhance either the proinflammatory or antiinflammatory immune responses. Th1/Tc1 cells are known to be pathogenic effector cells in T1D (37–39).

Precommitted Th2- and Th1-type cells influence polarization of T cells that are in close proximity, for example, during recirculation through inflamed tissue and lymph nodes (LNs). Within inflamed tissue precommitted cells are thought to commit to a specific lineage on encounter with specific antigen (40). To recirculate through LNs where primary responses to antigen generally take place (41), T cells must express homing receptors, CCR7 and CD62L, as well as the cell surface markers that distinguish naive and central memory (CM) T cells from effector memory (EM) T cells (42, 43). As cells differentiate from naive and CM to EM, expression of both CCR7 and CD62L is reduced, giving the cells access to peripheral tissue (42, 43). By combining cytokine profiles with the expression of cell surface markers the potential function of T cell subsets that are associated with clinical outcomes can be predicted. In our study, the frequency of the 127-hi cell population that is preferentially made up of IL-4–secreting pre-Th2 memory cells is associated with LoR and β cell function. We suggest that 127-hi cells are actively involved in delaying disease progression by inhibiting the expansion and differentiation of potentially pathogenic autoimmune Th1/Tc1 cells.

Retrospective analyses of clinical trial data show that patients with T1D are more likely to respond well to immunotherapy if it is given soon after diagnosis (3–6). However, even within those patients treated within 3 months of diagnosis, not all patients respond equally. This heterogeneity in response to immunotherapy is not fully understood (44). We suggest the hypothesis that, of the patients treated soon after diagnosis, those in partial remission will respond better to treatment, because the mechanism of the partial remission period provides a permissive environment for immunotherapy. Furthermore, we might expect that patients with the highest frequency of 127-hi cells, who are therefore predicted to have the longest partial remission and the highest level of protection, would respond best to immunotherapy. As part of this study we tested whether 127-hi cell frequency at diagnosis correlates with response to the antiinflammatory drug alefacept in patients with T1D recruited to the T1DAL study (2, 45, 46).

## Results

The source, number, sex, and age of the participants in all figures and tables are summarized in Table 1. For this study, the first time point for blood sample collection was within 3 months of diagnosis. Throughout, this time point is referred to as “baseline.”

### 127-hi cells are equivalently quantified at different research sites.

**Figure 1:**
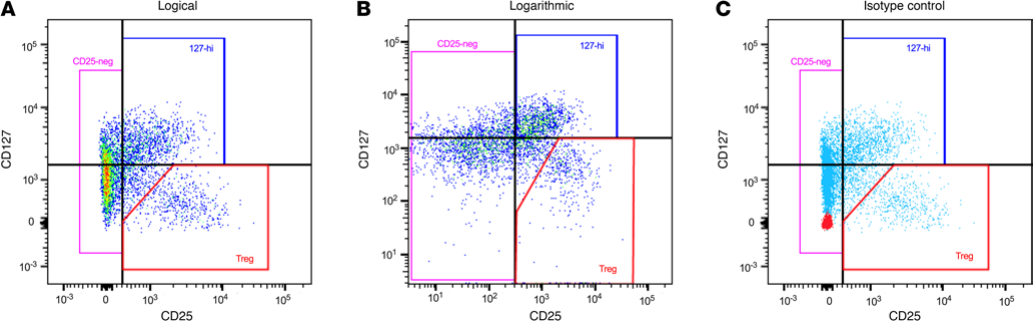
A schematic to identify 127-hi cells. Frozen PBMCs isolated from healthy adults were labeled for CD3, CD4, CD25, and CD127. Coexpression of CD25 and CD127 on gated CD3^+^CD4^+^ cells is presented in logical (**A**) and logarithmic (**B**) scale. (**C**) Isotype control staining for CD25 and CD127 expression is shown in red. For **A–C**, 127-hi cells are in the box in the top right quadrant and coexpress CD127 and CD25 (CD25^+^CD127^hi^), Tregs are in the box in the bottom right quadrant and express a low density of CD127 and a high density of CD25, and CD25^–^ (CD25-neg) cells are in the top and bottom left quadrants. The data are representative of more than 10 healthy donor PBMC samples.

**Figure 2:**
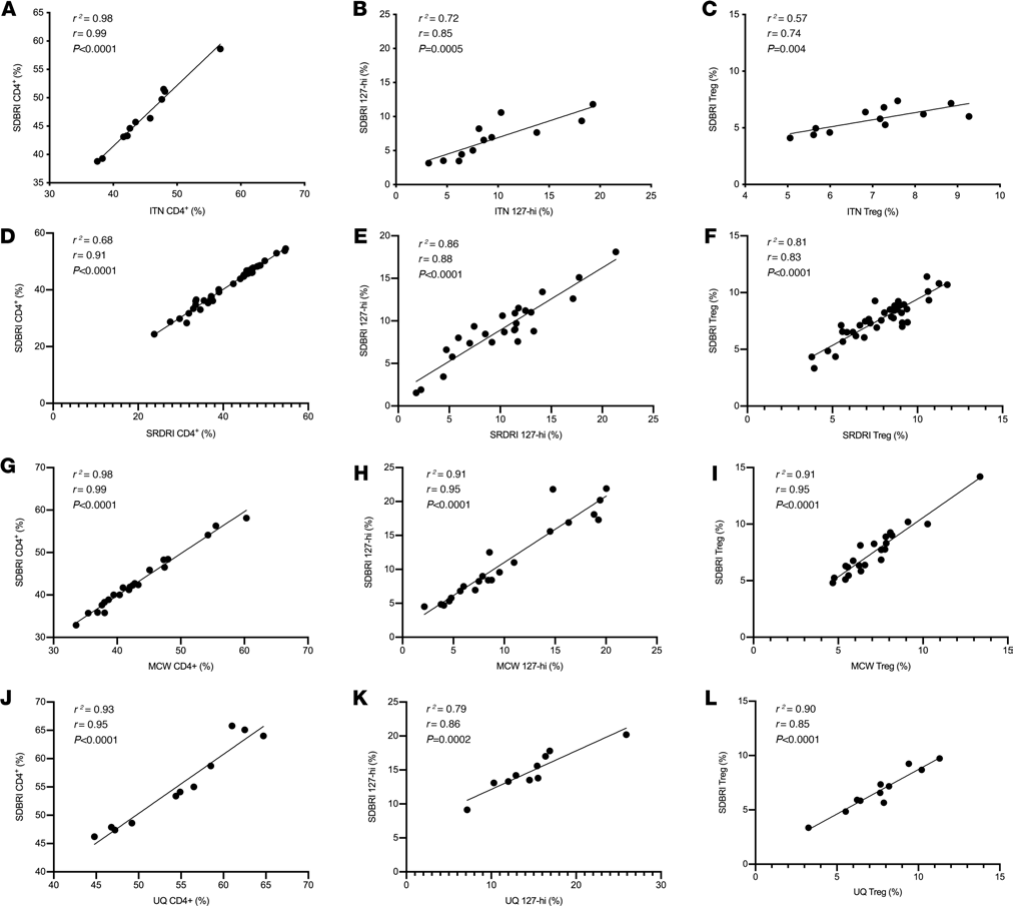
127-hi cells are equivalently quantified at different research sites. Flow cytometry data generated by investigators at ITN (**A**–**C**), SRDRI (**D**–**F**), MCW (**G**–**I**), and UQ (**J**–**L**) were used to determine whether the frequency of total CD4^+^ cells (**A**, **D**, **G**, and **J**), 127-hi cells (**B**, **E**, **H**, and **K**), and Tregs (**C**, **F**, **I**, and **L**) is equivalent when quantified by ITN and SDBRI (**A–C**), SRDRI and SDBRI (**D–F**), MCW and SDBRI (**G–I**), and UQ and SDBRI (**J–L**). The data were generated using PBMCs from patients with T1D. Samples were analyzed at baseline from the 12 patients at ITN (*n* = 12), 39 at SRDRI (*n* = 39), 22 at MCW (*n* = 22), and 11 at UQ (*n* = 11). PBMCs were labeled for CD3, CD4, CD25, and CD127. Each symbol represents an individual time point for each patient. The relationship between the relative frequency of each cell subset determined at different sites was determined using both Spearman’s correlation (*r*) and linear regression (*R*^2^). Values for *r* and *R*^2^ and *P* values are shown.

Investigators at all 4 research sites, ITN, SRDRI, MCW, and UQ, were asked to identify flow cytometry data from their own group generated using PBMCs from people within 3 months of diagnosis with T1D stained with a panel of antibodies that were specific for CD3, CD4, CD25, and CD127 (*n* = 12 for ITN, *n* = 39 for SRDRI, *n* = 22 for MCW, and *n* = 11 for UQ). Our group sent each collaborative group a schematic (Figure 1) that describes how to identify 127-hi cells, CD25^–^ cells, and Tregs. Each group was asked to record the relative frequency of 127-hi cells within the total CD4^+^ T cell population using their own raw flow cytometry data. The same data were blinded and sent to our group at San Diego Biomedical Research Institute (SDBRI) so that we could quantify the same cell subset using the same flow cytometry data. All investigators also quantified the relative frequency of established T cell populations, CD4^+^ T cells, and Tregs. The data were then exchanged and unblinded, and the frequency of 127-hi cells, CD4^+^ T cells, and Tregs in each sample was compared among investigators at different sites for consistency using linear regression (Figure 2). The mean fluorescence for CD25 and CD127 on 127-hi cells analyzed at the 4 different sites is shown in Supplemental Figure 1 (supplemental material available online with this article; https://doi.org/10.1172/jci.insight.136114DS1).

### The relative frequency of 127-hi cells at baseline correlates with LoR.

Data were pooled from ITN, SRDRI, MCW, and UQ to determine correlations between baseline measures of 127-hi cell frequency, age, BMI, insulin dose adjusted A1c (IDAA1c), and sex with LoR. When using Cox model single covariate analysis adjusted for study variability, 127-hi cell frequency at diagnosis correlated with LoR (*P* = 0.0227; *n* = 84), whereas age, BMI, baseline IDAA1c, and sex did not (*P* = 0.1929, *P* = 0.3323, *P* = 0.7029, *P* = 0.9790, respectively). Multivariate analysis also identified baseline 127-hi cell frequency as the only significant correlate with LoR (Table 2). However, the best fit model for correlation with LoR included 127-hi cell frequency with BMI (Table 3). Using a multivariate analysis add-back strategy to test the effect of BMI, age, sex, or baseline IDAA1c on the influence of 127-hi cell frequency on LoR, only BMI improved the correlation with LoR (Table 4).

To test whether there is a relationship between 127-hi cell frequency and age, BMI, or sex, in addition to IDAA1c, a linear regression of covariates was performed (Table 5). Baseline 127-hi cell frequency and age were significantly predictive of each other, as were baseline 127-hi cell frequency and BMI (Table 5). Moreover, at higher levels of BMI, the influence of age on 127-hi increased, and, at older age at diagnosis, the influence of BMI on 127-hi cell frequency increased (Table 6). Baseline IDAA1c and sex did not significantly predict 127-hi cell frequency (Table 5).

**Figure 3:**
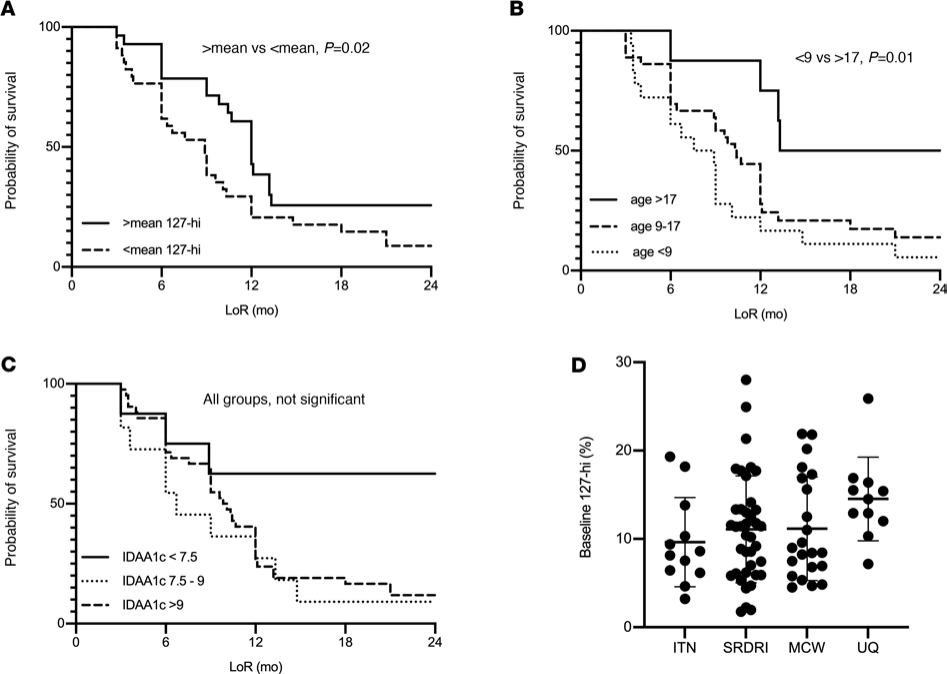
The probability of remaining in remission (survival probability) is greater in patients who have a higher baseline relative frequency of 127-hi cells and are older at diagnosis. The effect of the relative frequency of 127-hi cells (**A**), age (**B**), and IDAA1c level (**C**) at baseline on the probability of staying in remission was determined. Patients were stratified by (**A**) having a relative frequency of 127-hi cells either equal to or greater than the mean (*n* = 40, solid line) or lower than the mean (*n* = 44, dashed line); (**B**) by being older than 17 years at diagnosis (*n* = 8, solid line), or between 9 and 17 years (*n* = 50, dashed line), or younger than 9 years (*n* = 26, solid line); or (**C**) by having a baseline IDAA1c lower than 7.5 (*n* = 8, solid line), between 7.5 and 9 (*n* = 11, dotted line), or higher than 9 (*n* = 62, dashed line). Statistical significance was determined using log-rank (Mantel-Cox) test. For **A**, *P* = 0.02 for 127-hi cell frequency greater than the mean compared with below the mean. For **B**, *P* = 0.01 for the age group greater than 17 years of age compared with the group younger than 9 years; no significant differences between other groups. For **C**, no significant differences between groups. (**D**) Relative frequency of baseline 127-hi cells for all samples used in this figure. The log-rank test for trend was also used for **B** (*P* = 0.003) and (**C**) (*P* = 0.13, not significant). *P* < 0.05 is considered significant.

The effect of 127-hi cell frequency at baseline, age at diagnosis, and baseline IDAA1c on survival probability was also determined, where the limit of survival was the end of remission. End of remission occurred significantly earlier in patients with a baseline 127-hi cell frequency below the mean compared with those with a 127-hi cell frequency above the mean (Figure 3A; *P* = 0.02). To examine the effect of age, patients were stratified by being older than 17 years, between 9 and 17 years, and younger than 9 years at diagnosis. Patients diagnosed with T1D when they were older than 17 years had a significantly longer end of remission compared with children younger than 9 years (Figure 3B; *P* = 0.01). There was no significant difference in end of remission between the group aged between 9 and 17 years at diagnosis and either of the other groups. Using the log-rank test for trend, there was also a significant association between earlier end of remission in younger patients and longer end of remission in patients 17 years and older (Figure 3B; *P* = 0.003). The end of remission was not different in patients stratified by having an IDAA1c level at baseline of less than 7.5, between 7.5 and 9, or greater than 9 (Figure 3C). Figure 3D shows the relative frequency of 127-hi cells for all samples used in the analyses shown in Figure 3, A–C, from ITN, SRDRI, MCW, and UQ.

### The probability of preserving β cell function is greater in patients with a higher relative frequency of 127-hi cells at diagnosis.

**Figure 4:**
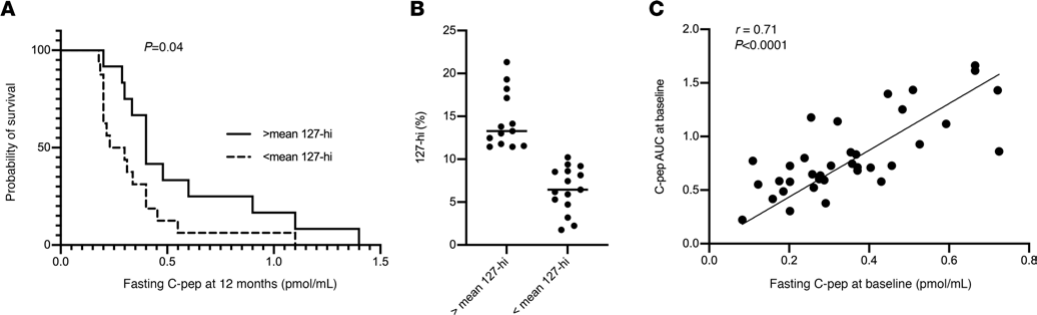
The probability of preserving β cell function is greater in patients with a higher relative frequency of 127-hi cells at diagnosis. (**A**) The relative frequency of 127-hi cells was determined in PBMCs collected by ITN (*n* = 9) and SRDRI (*n* = 19) at baseline from patients with T1D with good glucose control. Fasting C-peptide levels were measured in each patient at 12 months after diagnosis. Patients were stratified into groups based on having either equal to or greater than the mean frequency of 127-hi cells (*n* = 13, solid line) or lower than the mean frequency of 127-hi cells (*n* = 15, dashed line) at baseline. Statistical significance was determined using the log-rank (Mantel-Cox) test. (**B**) The relative frequency of 127-hi cells at baseline for patient data used in the analysis shown in **A**. Each symbol represents an individual patient. (**C**) In a different cohort of patients with good glucose control, fasting C-peptide levels and stimulated C-peptide AUC were measured at baseline and the correlation between the 2 C-peptide values was determined using Spearman’s correlation (*n* = 36).

We next tested the relationship between 127-hi cell frequency at baseline and future β cell function by measuring the effect of 127-hi cell frequency on probability of preserving β cell function, where the limit of survival was fasting C-peptide level at 12 months after diagnosis (Figure 4, A and B). The fasting C-peptide level at 12 months after diagnosis in patients with good glucose control was significantly lower in patients with a baseline 127-hi cell frequency below the mean compared with those with a 127-hi cell frequency above the mean (Figure 4A; *n* = 28; *P* = 0.04). However, when the analysis included all patients tested (*n* = 42), including those with poor glucose control (*n* = 14), the relationship was no longer significant.

Fasting C-peptide is less commonly used to assess β cell function than stimulated C-peptide. In our study, fasting C-peptide was the measurement of choice because stimulated C-peptide was measured in only 12 of the 84 participants, whereas fasting C-peptide was measured in 42 patients. To determine the relationship between levels of fasting and stimulated C-peptide, we compared these 2 measurements in the same blood samples collected at baseline. Only patients with good glucose control were included. The data showed a strong correlation between levels of fasting C-peptide and stimulated C-peptide AUC in this population (Figure 4C; *r* = 0.71, *P* < 0.0001).

### The relative frequency of 127-hi cells decreases over time after diagnosis.

**Figure 5:**
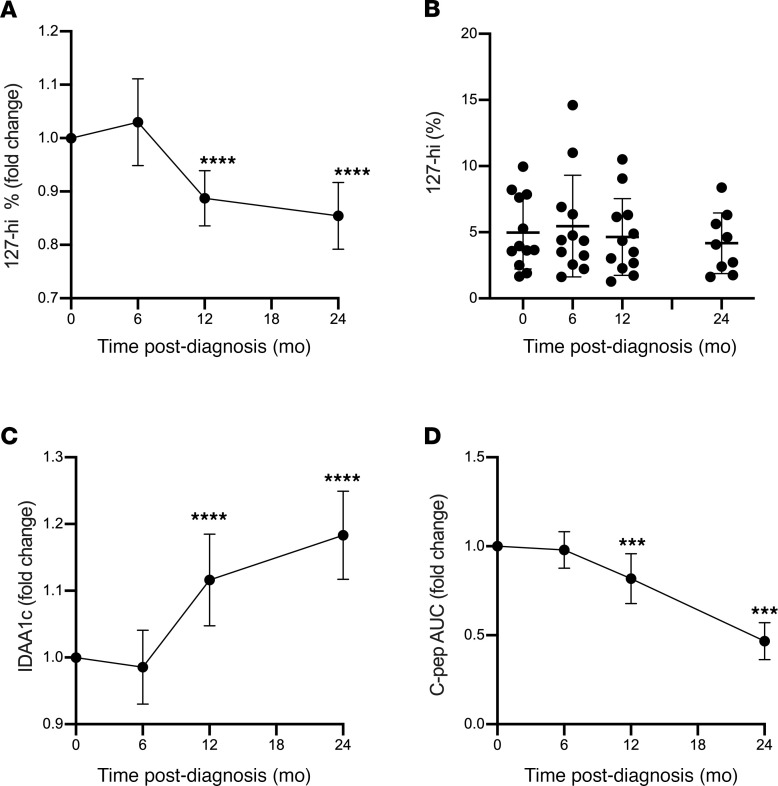
The relative frequency of 127-hi cells decreases with time after diagnosis. The frequency of 127-hi cells (**A** and **B**), IDAA1c levels (**C**), and stimulated C-peptide AUC (**D**) were determined at baseline (time 0 months) and at 6, 12, and 24 months after baseline (*n* = 12). The fold change for each parameter was compared with baseline using the paired Student’s *t* test. ***P = 0.0009–0.0001, ****P < 0.0001. (**B**) The relative frequency of each individual at each time point. *P* ≤ 0.05 is considered significant.

The relative frequency of 127-hi cells was determined at 6, 12, and 24 months and compared with baseline values to determine whether there is a decline in this cell population over time. Changes in IDAA1c and C-peptide levels were also determined. The relative frequency of 127-hi cells was reduced by 12 and 24 months after baseline (Figure 5, A and B). As expected, IDAA1c levels increased (Figure 5C) and C-peptide levels declined with time after diagnosis (Figure 5D).

### 127-hi cells from people with T1D are predominantly memory cells with a Th2 bias.

**Figure 6:**
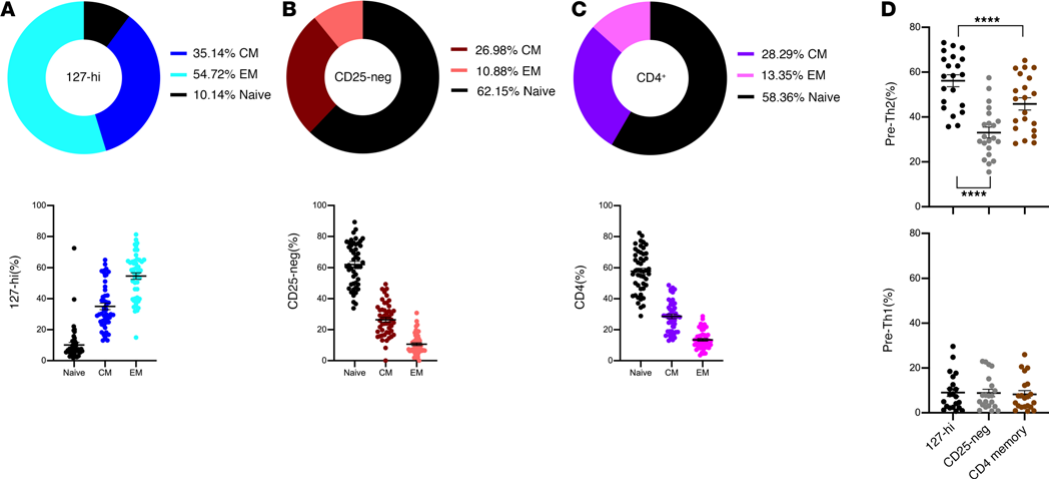
127-hi cells from people with T1D are predominantly memory cells with a Th2 bias. The frequency of naive, CM, and EM cells in 127-hi cells or CD25^–^ (CD25-neg) cells or total CD4^+^ T cells in PBMCs collected at baseline from patients with T1D was determined (*n* = 48). The pie charts show the mean of each subset within 127-hi (**A**), CD25^–^ (**B**), and total CD4^+^ (**C**) cells, and the bar graphs show the mean ± SEM for each cell subset within each population. (**D**) In a separate experiment, PBMCs from patients with T1D (*n* = 21) collected at baseline were evaluated for the relative frequency of precommitted Th2 cells and precommitted Th1 cells (**D**) within 127-hi memory cells, CD25^–^ memory cells, and CD4^+^ memory cells. Statistical differences between groups was calculated using ANOVA (*P* < 0.0001) followed by Sidak’s multiple comparison test. Data are shown as mean ± SEM. ****P < 0.0001.

In healthy individuals 127-hi cells are a mixture of naive, CM, EM, and precommitted Th1 and Th2 cells (21). To determine whether 127-hi cells from patients with T1D have the same mixed phenotype, the relative frequency of naive, CM, and EM cells within the 127-hi cell compartment in PBMCs from patients with T1D was determined. The same cell subsets were also quantified in the CD25^–^ and total CD4^+^ T cell compartments. More than 85% of 127-hi cells were memory cells, and the majority of these were EM (Figure 6A). In contrast, within the CD25^–^ (Figure 6B) and total CD4^+^ (Figure 6C) cell populations, more than 50% of the cells had a naive cell phenotype, and, of the memory phenotype cells, CM were in the majority (Figure 6C). We further characterized the 127-hi memory cells from patients with T1D by quantifying the relative frequency of pre-Th2 (CXCR5^–^CXCR3^–^CCR4^+^) and pre-Th1 (CXCR5^–^CXCR3^+^CCR4^–^) cells and comparing these frequencies with CD25^–^ and total CD4^+^ memory T cells. As seen in healthy people, 127-hi memory cells had a significantly higher pre-Th2 frequency than either the CD25^–^ or the total CD4^+^ memory cell populations, whereas the frequency of pre-Th1 cells was not different between the 127-hi and CD25^–^ memory cell compartments (Figure 6D).

### 127-hi pre-Th2 memory cells secrete significantly more Th2-type cytokines than CD25^–^ memory cells.

**Figure 7:**
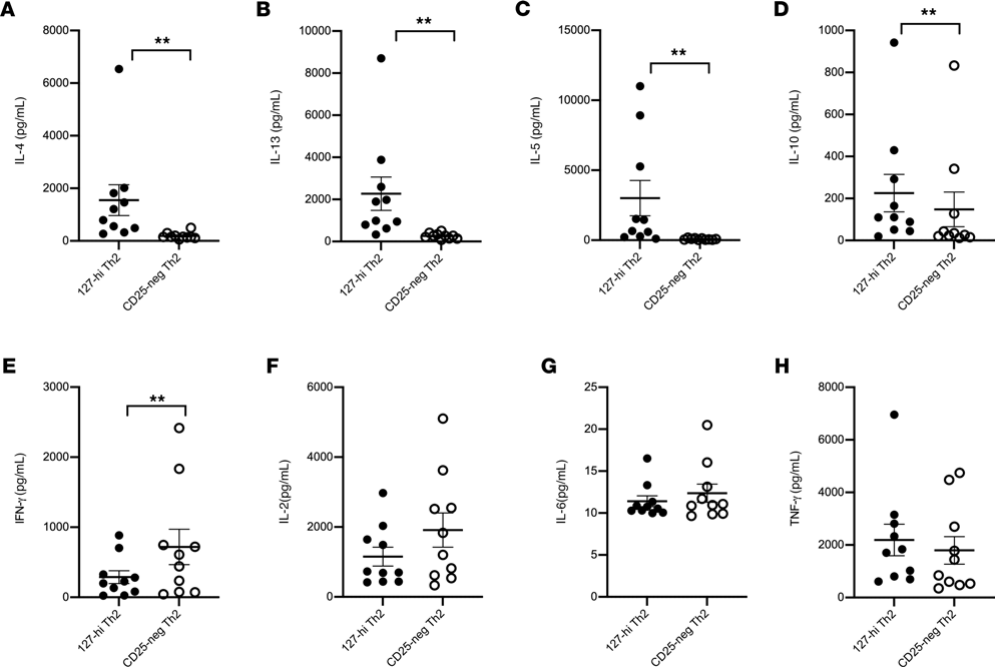
127-hi pre-Th2 memory cells secrete significantly more Th2-type cytokines than CD25^–^ memory cells. PBMCs collected at baseline from patients with T1D (*n* = 10) were labeled for CD3, CD4, CD45RO, CXCR5, CXCR3, CCR4, CD25, and CD127, and the 127-hi pre-Th2 (black circles) and CD25^–^ pre-Th2 (white circles) cells were sorted. Cells were stimulated with anti-CD3/anti-CD28 beads for 48 hours and supernatants were harvested. IL-4 (**A**), IL-13 (**B**), IL-5 (**C**), IL-10 (**D**), IFN-γ (**E**), IL-2 (**F**), IL-6 (**G**), and TNF-α (**H**) levels were measured. Statistical significance was calculated using Wilcoxon’s matched-pairs signed-rank test. Data are shown as mean ± SEM. **P = 0.009–0.001.

The capacity of 127-hi and CD25^–^ pre-Th2 memory cells to secrete Th2-type cytokines was tested by sorting pre-Th2 127-hi memory and pre-Th2 CD25^–^ memory cells and measuring Th2- and Th1-type cytokines after stimulation for 48 hours in vitro. 127-hi pre-Th2 cells secreted significantly more IL-4, IL-13, IL-5, and IL-10 (Figure 7, A–D) and significantly less IFN-γ than CD25^–^ pre-Th2 cells (Figure 7E). Levels of IL-2, IL-6,and TNF-α were not different between groups (Figure 7, F–H). As part of the same experiment pre-Th1 memory cells were also sorted. However, insufficient numbers of cells were available for analysis (Supplemental Table 1).

### The 127-hi cell population contains significantly more GATA-3^+^ cells and fewer T-bet^+^ cells than CD25^–^ cells.

**Figure 8:**
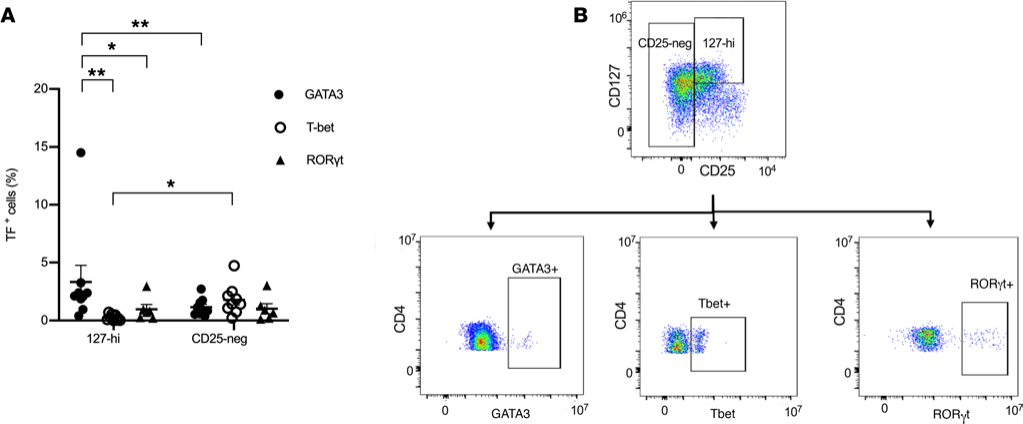
The 127-hi cell population contains significantly more GATA-3^+^ cells and fewer T-bet^+^ cells than CD25^–^ cells. PBMCs from patients with T1D, collected at baseline, were stimulated for 4 hours with PMA and ionomycin. Stimulated cells were labeled for CD3, CD4, CD45RO, CD25, and CD127 and intracellular GATA-3 (black circles, *n* = 9), T-bet (white circles, *n* = 9), or RORγt (black triangles, *n* = 6). (**A**) Relative frequency of GATA-3, T-bet, and RORγt in either 127-hi cells or CD25^–^ cells. (**B**) Coexpression of CD127 and CD25 on CD3^+^CD4^+^CD45RO^+^ memory cells. Rectangles show the gating strategy to identify either CD25^–^ or 127-hi cells. Representative plots showing expression of GATA-3, T-bet, and RORγt in 127-hi and CD25^–^ cells. Statistical significance in **A** was calculated using Welch’s ANOVA (*P* = 0.003) and Wilcoxon’s matched-pairs signed-rank test. Data are shown as mean ± SEM. *P = 0.05–0.01, **P = 0.009–0.001.

PBMCs from patients with T1D, collected at baseline, were stimulated with PMA and ionomycin for 4 hours. After stimulation cells were labeled for cell surface CD3, CD4, CD45RO, CD25, and CD127; intracellular expression of GATA-3, T-bet, and RORγt and transcription factor expression in 127-hi memory cells was compared with that in CD25^–^ cells. The frequency of 127-hi memory cells that express GATA-3 was significantly higher than the frequency of 127-hi cells that expressed either T-bet or RORγt (Figure 8A). In addition, the frequency of GATA-3^+^ cells was significantly higher in 127-hi cells than in CD25^–^ cells, whereas the frequency of T-bet^+^ cells was higher in CD25^–^ cells (Figure 8A). The frequency of RORγt^+^ cells was not different in the 2 cell subsets. Figure 8B shows representative expression of GATA-3, T-bet, and RORγt. No correlation was seen between the relative frequency of 127-hi cells that expressed GATA-3, T-bet, or RORγt and the relative frequency of 127-hi cells (Supplemental Figure 2).

### Treatment with alefacept depletes circulating 127-hi EM cells.

**Figure 9:**
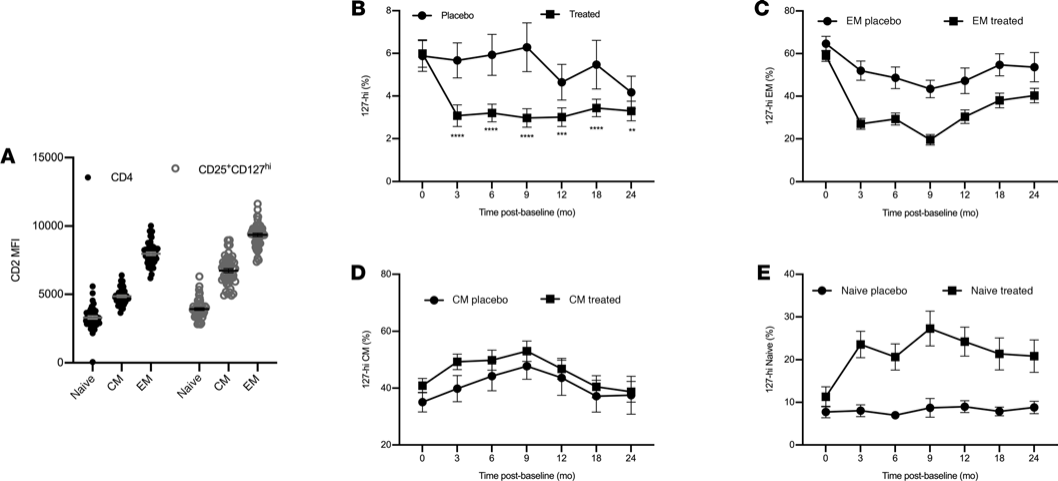
Treatment with alefacept depletes circulating 127-hi EM cells. (**A**) Data generated from T1DAL study participants by ITN using PBMCs collected at baseline (before treatment began) from both the placebo and treated groups were reanalyzed at SDBRI to determine the level of cell surface expression of CD2 on 127-hi EM, CM, and naive cells compared with EM, CM, and naive cells in the total CD4^+^ T cell population, respectively (*n* = 48). The relative frequency of total 127-hi cells (**B**), 127-hi EM cells (**C**), 127-hi CM cells (**D**), and 127-hi naive cells (**E**) was determined at either 3- or 6-month intervals from baseline to 2 years after baseline in the T1DAL study treated (*n* = 33) and placebo (*n* = 16) groups. Analysis of data shown in **B** was performed using repeated-measures ANOVA for months 3, 6, 9, 12, 18, and 24. **P = 0.009–0.001, ***P = 0.0009–0.0001, ****P < 0.0001.

Alefacept was originally used as an antiinflammatory drug for patients with psoriasis (47–50). Alefacept works by inhibiting CD2-mediated costimulation and by depleting CD2-expressing cells (51, 52). Because CD2 is expressed at a high level on EM cells, which are known to cause pathology in T1D, alefacept was tested, with some success, as an immunotherapy to reverse T1D (2, 45, 46). Further characterization of 127-hi cells showed that 127-hi EM cells express a higher density of CD2 than do 127-hi naive and CM cells (Figure 9A), identifying 127-hi EM cells as a potential target for alefacept. In the T1DAL clinical trial, treated patients with T1D were given two 12-week courses of alefacept, the first beginning at baseline and the second beginning at 24 weeks after baseline. Our data show that alefacept significantly depleted 127-hi cells after both the first (tested at 3 months after baseline) and second round of treatment (tested at 9 months after baseline; Figure 9B). 127-hi EM cells are the main target for alefacept, but they appeared to recover quickly after the second treatment was completed (Figure 9C). In contrast, the frequency of CM cells remained unaffected (Figure 9D). The relative frequency of naive cells increased as EM frequency decreased (Figure 9E).

### The probability of remaining in remission (survival probability) is greater in alefacept-treated patients who have a higher baseline relative frequency of 127-hi cells.

**Figure 10:**
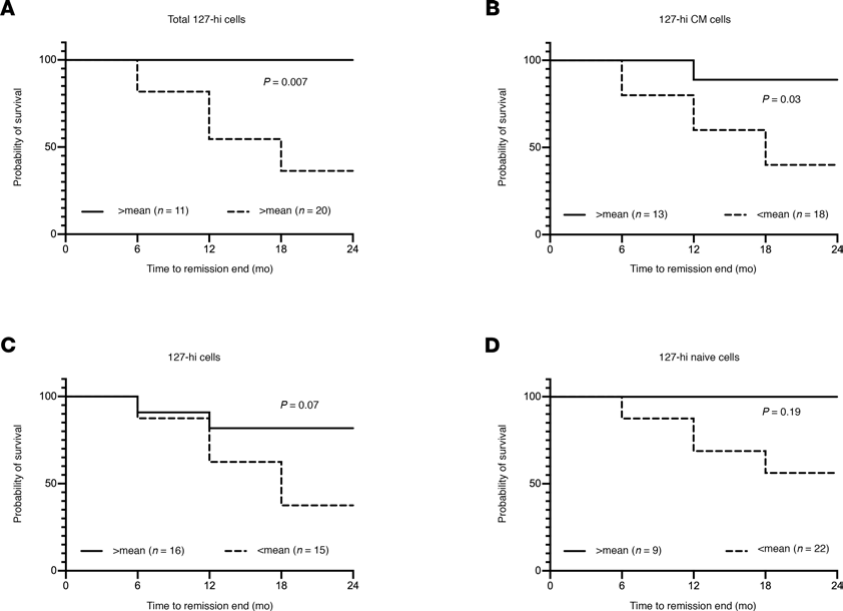
The probability of remaining in remission (survival probability) is greater in alefacept-treated patients who have a higher baseline relative frequency of 127-hi cells. Patients were stratified based on having either lower than the mean (dashed line) or equal to or greater than the mean (solid line) relative frequency of total 127-hi cells (**A**, *n* = 11 > mean, *n* = 20 < mean), 127-hi CM cells (**B**, *n* = 13 > mean, *n* = 18 < mean), 127-hi EM cells (**C**, *n* = 16 > mean, *n* = 15 < mean), and 127-hi naive cells (**D**, *n* = 9 > mean, *n* = 22 < mean) at baseline. Statistical significance was determined using the log-rank (Mantel Cox) test.

We compared the probability of remaining in remission in alefacept-treated patients with a baseline frequency of 127-hi cells above the mean compared with a frequency below the mean. We found that those above the mean were significantly more likely to remain in remission at 2 years after baseline compared with those below the mean (Figure 10A). The same was true for 127-hi CM cells (Figure 10B) but not for 127-hi EM cells (Figure 10C) or 127-hi naive cells (Figure 10D).

## Discussion

This study validates our previously published finding that the probability of remaining in remission for patients newly diagnosed with T1D is significantly greater for the patients who have the highest relative frequency of 127-hi cells at diagnosis (19). In addition, we extend these findings to show that patients with the highest frequency of 127-hi cells have the greatest probability of preserving β cell function. Furthermore, as β cell function declines over time after diagnosis, 127-hi cell frequency is reduced, again associating with disease progression. We suggest that, if 127-hi cells are mechanistically involved in promoting and maintaining partial remission, a reduction in their frequency as β cell function declines might be expected. As seen previously for 127-hi cells in healthy people (21), 127-hi cells in patients with T1D are a mix of naive, CM, and EM, pre-Th1 and pre-Th2 cells, with a bias toward Th2. The Th2 bias suggests the possibility that 127-hi cells play an active role in prolonging partial remission by deviating potentially pathogenic Th1-type cells toward the antiinflammatory Th2-type. In addition, the potential for 127-hi cell frequency to predict response to immunotherapy is suggested by the finding that patients with a higher baseline 127-hi cell frequency have a greater probability of remaining in remission at 2 years after diagnosis after treatment with the antiinflammatory drug alefacept.

We first identified 127-hi cells by their expression of CD25 with a high density of CD127 (19). These cells also express a high density of CD44 and CD44v6 (19). The finding that 127-hi cells can be equivalently identified and quantified by different research groups validates the 127-hi cell population as a measurable component of the CD4^+^ T cell compartment. The frequency of circulating 127-hi cells in patients with T1D might be controlled by their expression of CD25, CD127, CD44, and CD44v6. Thus, CD25- and CD127-mediated signaling after binding to circulating IL-2 and IL-7, respectively, promotes the survival and expansion of T cell populations (53). However, in inflammatory environments, including in patients with T1D, activated T cells shed CD25 and CD127 into the circulation where they bind their respective cytokines, lowering the concentration of circulating IL-2 and IL-7 (54–56). During hyperglycemia, sCD127 is glycated making it inaccessible to IL-7 and ineffective as an inhibitor of IL-7 signaling (54). CD44 and CD44v6 are receptors for the extracellular matrix component hyaluronan, signaling through which induces CD25 expression (57), inhibiting cell apoptosis (58–60). However, during inflammation, hyaluronan breaks down into low-molecular-weight forms that fail to cross link CD44 and CD44v6 (61). Taken together these data suggest that the level of 127-hi cells in patients with T1D reflects the level of inflammation.

The Th2 bias of 127-hi cells was originally reported in healthy people (21) and shown again here in patients with T1D. 127-hi cells in healthy people (21) and in patients with T1D, shown in this study, secrete significantly higher quantities of the Th2 cytokine IL-4 than other CD4 memory cells, giving them the potential to be more potent inhibitors of pathogenic Th1/Tc1-type responses. This Th2 bias, combined with the knowledge that a higher frequency of 127-hi cells correlates with longer partial remission and preserved β cell function, suggests that 127-hi cells might play an active role in delaying disease progression by inhibiting the expansion of Th1 cells via Th2 cell–mediated immune deviation (22–24). 127-hi cells might also regulate disease progression via their expression of CD44v6, signaling through which induces the expression of Foxp3, IL-2, TGF-β, and IL-10 (57, 58), factors that can promote both Tregs and Tr1 cells (62, 63). This provides additional potential mechanisms by which 127-hi cells might protect from disease progression.

Immune deviation by Th2 cells and immune regulation by Tregs and Tr1 cells require close proximity between the regulatory cell and the effector cells. Such regulatory function takes place in lymphoid organs, including LNs, where T cells priming takes place (41), and in the peripheral tissue (40). CM cells, including 127-hi CM cells, express the homing receptors CCR7 and CD62L, allowing them to circulate between the blood and lymphatic system via LNs (42, 43). It is possible that while in the LN 127-hi CM cells create a Th2-type microenvironment capable of deviating the differentiation of primary autoantigen-reactive T cell responses toward the less inflammatory Th2 type and away from the proinflammatory Th1-type.

Previously published studies have shown significant associations between age (11, 64–68) and partial remission. Consistent with these findings, we show that the probability of remaining in remission (survival probability) is greater in patients who are older at diagnosis. However, when we use multivariate analysis to test the relative strength of the relationships between age and sex, 127-hi cell frequency, BMI, and baseline IDAA1c with LoR and when all of these parameters are combined, 127-hi cell frequency is the only covariate that predicts LoR. Moreover, 127-hi cell frequency is a significant predictor of age and vice versa, which is consistent with the notion that 127-hi cell frequency and function might explain the association between partial remission and age.

Consistent with the concept that partial remission is actively controlled by a form of immune regulation, the duration of persistent insulin secretion is negatively related to levels of inflammation and positively associated with abundances of circulating activated Tregs measured near clinical onset (69). Moreover, several investigators have investigated the possibility that levels of circulating cytokines, chemokines, other molecules, and immune cell subsets can be used as biomarkers to predict partial remission. Elevated levels of IL-6, a Th2-type cytokine that is proinflammatory when in its bound to soluble IL-6 receptor and antiinflammatory when not (70), is positively associated with remission (71). Elevated levels of IL-10, also a Th2-type cytokine, are also positively associated with remission (72), whereas serum levels of IFN-γ are negatively associated with remission (73). It is possible that levels of these cytokines reflect a change in the frequencies of circulating 127-hi Th2 and Th1 cells. The frequency of apoptotic Tregs is negatively related to remission (74). Although these associations are significant, none are sufficiently accurate to be considered biomarkers. Similarly, although baseline 127-hi cell frequency is significantly associated with LoR and the probability of remaining in remission and preserving β cell function, suggesting a mechanistic link, the associations are not sufficiently strong for 127-hi cell frequency to be used as a biomarker for clinical outcome. An additional advantage to better understanding the mechanism of partial remission is that markers that accurately reflect that mechanism might be used as biomarkers to predict the beginning, duration, and end of the remission period.

Alefacept is an LFA-3 dimer fused to the Fc portion of IgG1 and was first used as an antiinflammatory drug to treat psoriasis (47–50). Alefacept depletes its target cells by binding to CD2, which is expressed at different levels on EM, CM, and naive T cells in the order EM > CM > naive. Those cells that express the highest density are depleted the most effectively (51, 52). Because EM cells are a known pathogenic effector cell in T1D, alefacept was also identified as a treatment for T1D (2, 45, 46). Alefacept did not significantly delay T1D progression in the treated group as a whole compared with the placebo group; however, it did preserve β cell function in several patients for at least 2 years (2). Retrospective analysis of flow cytometry data shows that alefacept depletes both EM and CM cells but that EM cells were depleted more efficiently (2). In contrast, Tregs were not depleted (2). Reanalysis of these data by our group revealed that 127-hi cells, preferentially 127-hi EM cells, were also significantly depleted, but there was little evidence of an effect on 127-hi CM and 127-hi naive cells. Moreover, further analysis showed that the probability of remaining in remission after alefacept treatment was significantly greater in those patients with the highest frequency of both total 127-hi cells and 127-hi CM cells at baseline, but there was no relationship with 127-hi EM and 127-hi naive cells. These data suggest that the correlation between 127-hi cells and protection from disease progression is linked to either the 127-hi cell population as a whole or to CM cells.

Further research will expose the cellular and molecular pathways that drive the association between 127-hi cells and partial remission, verify whether 127-hi cells play a role in prolonging the duration of remission, and elucidate the central mechanism. Elements within mechanistic pathways that explain the relationship between 127-hi cells and disease progression might be used as biomarkers to select the patients most likely to respond favorable to immunotherapy.

## Methods

### Patient population.

Flow cytometry data and samples for this study were obtained from patients from 4 different sources: ITN, SRDRI, MCW and UQ. Table 1 shows the source of patient data and samples described in Figures 2–10 and Tables 2–4. Prior to this collaborative study, PBMCs from the patients with T1D recruited by ITN, SRDRI, MCW, and UQ were labeled at the respective collaborator laboratories with monoclonal antibodies specific for CD3, CD4, CD25, and CD127 to identify CD4^+^ Foxp3^+^ Tregs using flow cytometry. Tregs were identified by their expression of CD3, CD4, and CD25 and a low density of CD127 (28). The 127-hi cell population was identified with the same antibody panel used to identify Tregs, except that 127-hi cells were identified by their expression of CD3, CD4, and CD25 and a high density of CD127 (19). For the same patients, each collaborator site also measured and recorded insulin dose, HbA1c, and C-peptide levels at either 3- or 6-month intervals for at least 2 years.

### Healthy subject population.

Whole blood from healthy donors was obtained by SDBRI from the Normal Blood Donor Program at The Scripps Research Institute (TSRI). Whole blood was collected in heparin and processed within 2 hours. PBMCs were isolated using standard methods and frozen in liquid nitrogen.

### Measurement of partial remission and β cell function using IDAA1c and C-peptide AUC.

A standard formula, HbA1c (%) + 4× insulin dose (U/kg per 24 hours), was used to take into account both insulin requirement and HbA1c levels in a single value, the IDAA1c. An IDAA1c equal to or less than 9 indicates the partial remission period (75). In this study, the end of partial remission was between the last visit when IDAA1c was equal to or less than 9 and the first visit when IDAA1c was greater than 9. Length of remission was defined as the time between diagnosis and end of remission. Stimulated C-peptide AUC was calculated over 120 minutes using the trapezoidal rule, with observed C-peptide values at 0, 15, 30, 60, 90, and 120 minutes. Fasting C-peptide was measured in pmol/mL.

### Good and poor glucose control.

For some experiments, patients were stratified for analysis into those with either good glycemic control, defined as having a LoR longer than 1 month, or poor glycemic control, defined as having a LoR shorter than 1 month.

### T cell subsets identified by flow cytometry.

Fluorochromes, vendors, catalog numbers, and registry identifiers for all antibodies used in flow cytometry experiments, including isotype control antibodies, are as shown in Supplemental Table 1. Vials of PBMCs from patients with T1D were thawed and stained with the antibody panels listed in Supplemental Table 2. Healthy subject PBMCs were thawed and used as positive controls for mAb staining in each experiment.

To identify CD4^+^, 127-hi, CD4^+^CD25^–^ (CD25^–^) T cells, and CD25^+^CD127^lo^ (Tregs), we used either the BD Biosciences Treg cocktail or individual antibodies for CD3, CD4, CD25, and CD127 (see Supplemental Table 2). The MFI for CD3, CD4, CD25, and CD127 on 127-hi cells, CD25^–^ cells, and Tregs analyzed at each of the 4 collaborative sites is shown in Supplemental Figure 1. No differences were seen in the relative frequency of 127-hi cells, CD25^–^ cells, and Tregs from thawed PBMCs compared with freshly isolated PBMCs (Supplemental Figure 3). The relative frequency of naive (CD45RA^+^, CD45RO^–^, CCR7^+^), CM (CD45RA^–^, CD45RO^+^, CCR7^+^), and EM (CD45RA^–^, CD45RO^+^, CCR7^–^) cells in total CD4^+^, 127-hi, and CD25^–^ cells was determined by cell surface phenotype using anti-CCR7, anti-CD45RA, and anti-CD45RO. Tregs were identified as CD3^+^CD4^+^CD25^hi^CD127^lo^ as previously described (28). To identify Th1 and Th2 cell subsets within CD4^+^, 127-hi, and CD25^–^ memory cell populations, PBMCs were also labeled for CD45RO, CXCR5, CXCR3, and CCR4 to identify precommitted Th1 (CD45RO^+^CXCR5^–^CXCR3^+^CCR4^–^) and precommitted Th2 (CD45RO^+^CXCR5^–^CXCR3^–^CCR4^+^) (76, 77) memory cells. To identify cells that express transcription factors for the Th2 (GATA-3), Th1 (T-bet), or Th17 (RORγt) cells subsets, PBMCs were washed twice in RPMI (Invitrogen) with 10% human AB serum and rested at 37°C overnight. Cells were resuspended in RPMI with 10% human AB serum, HEPES (Gibco BRL), glutamine, penicillin, streptomycin (Irvine Scientific), and 2-mercaptoethanol (MilliporeSigma) and cultured in 24-well plates at a concentration of 1 × 10^6^ to 3 × 10^6^ cell per ml with 50 ng/ml PMA (MilliporeSigma) and 1 μM Ionomycin (MilliporeSigma). 1 μl Brefeldin A (BD Bioscience) per ml medium was added at the beginning of the culture. After 4 hours, cultured cells were washed twice. Intracellular expression of GATA-3, T-bet, and RORγt was measured. Data were acquired on an LSR II (BD, used at ITN), FACSCanto II (BD, used at SRDRI), LSR Fortessa and LSR II (both BD, used at MCW), LSRFortessa X20 (BD, used at UQ), and LSRFortessa (BD) or CytoFlex S (Beckman Coulter) (used at SDBRI) and analyzed using FlowJo version 10. Isotype controls were used in every experiment and for every antigen-specific antibody.

### Cell subset purification by sorting.

Pre-Th1 and pre-Th2 CD25^–^ memory and CD25^+^CD127^hi^ memory cells were identified using the antibodies described in the section above. The 4 cell populations were sorted on a BD FACSAria high-speed cell sorter. Gates used to sort cell subsets are shown in Supplemental Figure 4.

### Measurement of T cell cytokine secretion.

Sorted T cell subsets were incubated at 5000 cells per well in 96-well plates for 48 hours with 2 μl per well anti-CD3/CD28 coated beads (BD Biosciences), 10% human serum (Gemini), HEPES (Gibco BRL), glutamine, penicillin, streptomycin (Irvine Scientific), and 2-mercaptoethanol (MilliporeSigma) in RPMI (Invitrogen). Different plates were set up for different time points. After 48 hours of culture, 100 μl culture supernatant was collected and concentrations of IL-2, IL-4, IL-5, IL-6, IL-10, IL-13, IFN-γ, and TNF-α were determined by Flow Cytometry and the Biolegend LegendPlex Human Th2 panel.

### Statistics.

The relationship between the relative frequency of cell subsets determined at different research sites was determined using both Spearman’s correlation and linear regression. Associations between T cell subsets and probability of remaining in remission and the probability of preserving β cell function were assessed using the log-rank (Mantel-Cox) test and the log-rank test for trend. Univariate and multivariate Cox proportional hazards regression analyses were also used to assess the relationship between T cell subsets and clinical variables with LoR as the outcome variable. Covariates included the 127-hi cell subset frequency at baseline (time 0), age at diagnosis, sex, baseline IDAA1c, and BMI. These results are reported as hazard ratios, with 95% confidence intervals. Multivariate analysis was performed for up to 5 variables and adjusted for study variability. The relationship between covariates was determined with linear regression. The relationship between levels of fasting C-peptide and stimulated C-peptide AUC was determined using Spearman’s correlation. Changes in 127-hi cell frequency and IDAA1c and C-peptide levels at 6, 12, and 24 months compared with baseline were determined using the paired 2-tailed Student’s *t* test. One-way ANOVA followed by Sidak’s multiple comparison test was used to compare Th2/Th1 ratios between more than 2 cell populations. Differences in cytokine secretion between cells subsets was determined using Wilcoxon’s matched-pairs signed-rank test. Differences in transcription factor expression were calculated using Welch’s ANOVA (*P* = 0.003) and Wilcoxon’s matched-pairs signed-rank test. The effect of alefacept on the frequency of circulating 127-hi cells over 24 months was assessed using repeated-measures ANOVA. All analyses were performed with Graphpad Prism and SPSS V21.0 (IBM Corporation, 2012). A *P* value of less than 0.05 was considered statistically significant.

### Study approval.

All data reported in this study were collected by each collaborative organization as part of studies conducted prior to the study described, generated at SDBRI by reanalysis of previously generated data, or generated at SDBRI from staining vials of frozen PBMC samples obtained from ITN and collected by ITN as part of their prior studies. The sample and data analysis completed specifically for this study was approved by the SDBRI IRB, under exemption 4. Human subject protocol and consent forms were reviewed and approved by the TSRI IRB and the SDBRI IRB.

## Author contributions

AN generated the data for Figure 6, reanalyzed data for all figures and tables, and contributed to data analysis and to the writing of the manuscript. BL contributed to data generated for Figures 7 and 8. RM contributed to data generated for Figure 1. TTL analyzed data shown in Figures 3–5 and 9–10 and Tables 2–4. AM contributed to flow cytometry data analysis for Figure 2. IG and BP contributed to clinical data collection for patients from SRDRI. GG collected clinical samples and performed flow cytometry staining. RG contributed to data generated for Figure 3 and Tables 2–4. ASB, KB, and MH contributed to data used in Figure 3 and Figure 6D and Tables 2–4. RT provided data from UQ. MJH provided data from MCW. MB contributed to identifying the clinical collaborators and provided data from SRDRI. ES coordinated the transfer of data and samples from ITN and contributed to the design of statistical analysis and to the writing of the manuscript. JDD designed the research study and coordinated the collaborative team, samples, and data. Additionally, JDD analyzed the data and wrote the manuscript. All authors reviewed and approved the final manuscript.

## Supplementary Material

Supplemental data

## Figures and Tables

**Table 1:**
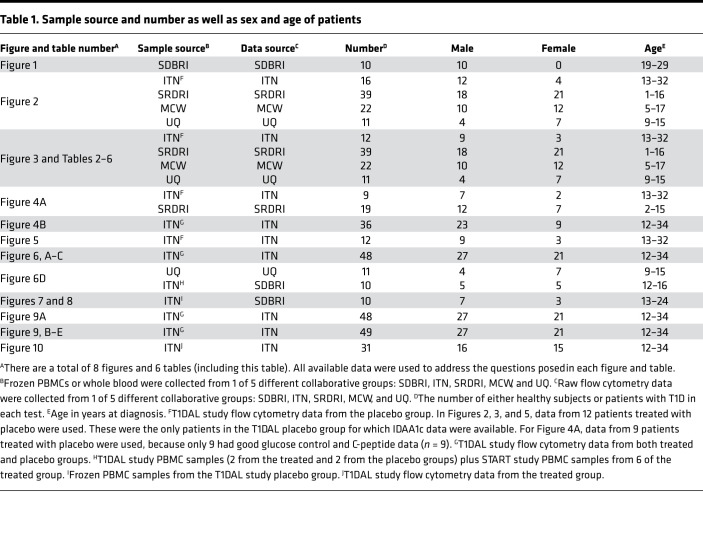
Sample source and number as well as sex and age of patients

**Table 2:**
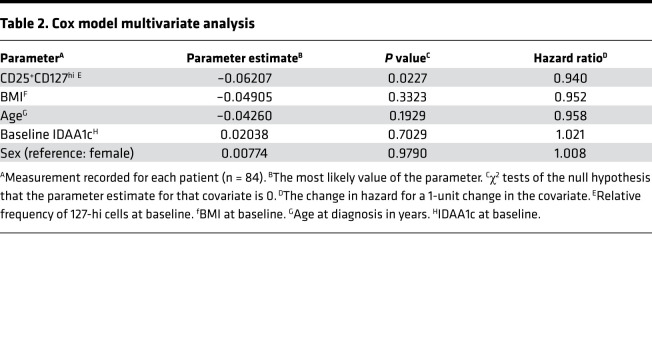
Cox model multivariate analysis

**Table 3:**
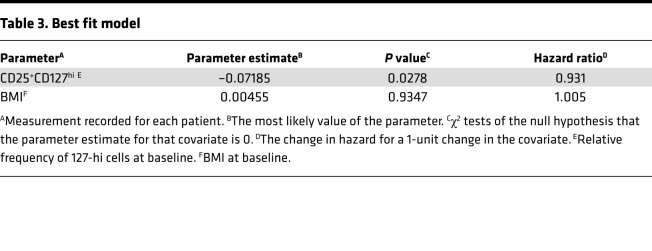
Best fit model

**Table 4:**
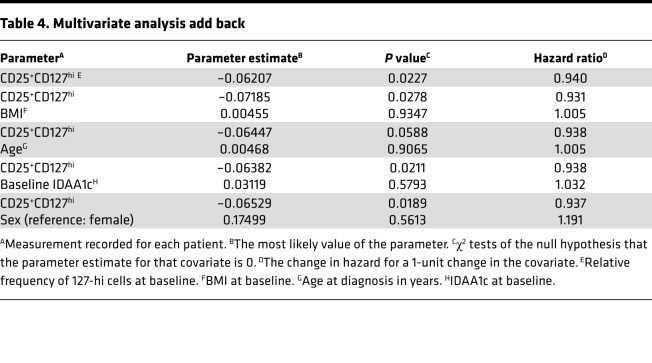
Multivariate analysis add back

**Table 5:**
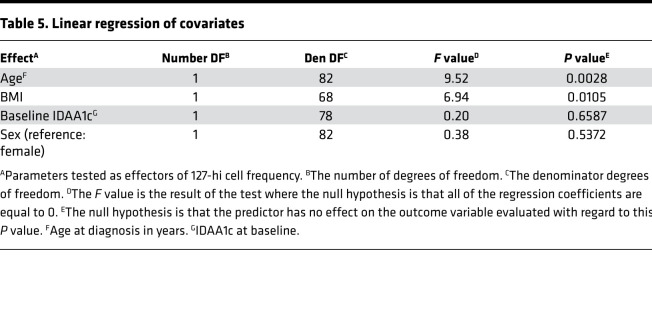
Linear regression of covariates

**Table 6:**
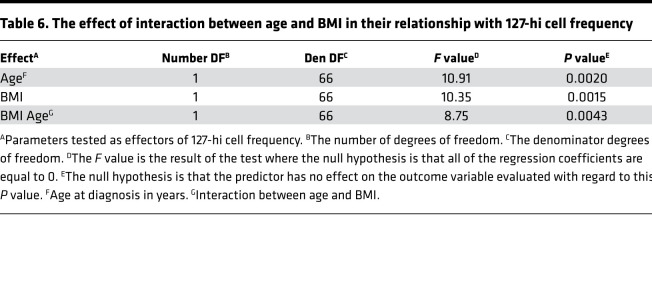
The effect of interaction between age and BMI in their relationship with 127-hi cell frequency

## Version 1 12/10/2020

In-Press Preview

## Version 2 01/25/2021

Electronic publication
