# Supplementary material for: CD4+CD25+CD127hi cell frequency predicts disease progression in type 1 diabetes
Source: JCI Insight. 2021 Jan 25;6(2):e136114. doi: 10.1172/jci.insight.136114 (PMC7934872; doi:10.1172/jci.insight.136114)
Supplement: Supplemental data [file jciinsight-6-136114-s195.pdf]

Supplemental Data

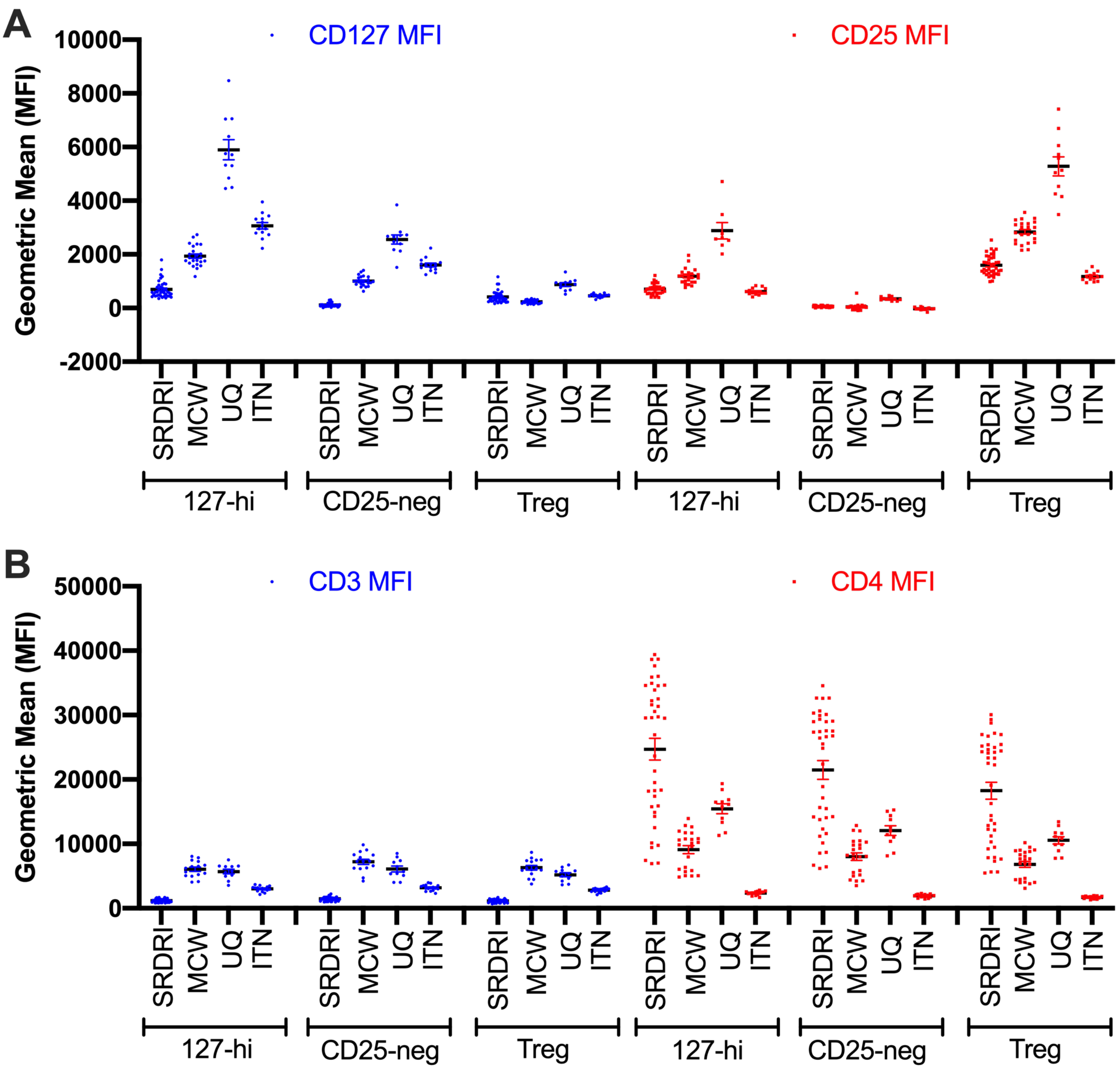

Supplemental Figure 1. The MFI for CD25 and CD127 on 127-hi cells using data generated at each site 127-hi cells were identified as shown in Figure 1. (A) shows the geometric mean fluorescence intensity (MFI) for 127 and CD25 on 127-hi cells, CD25-neg cells and Treg at SRDRI, MCW, UQ and ITN. (B) The MFI of CD3 and CD4 on the same cells at each of the four sites. The FSC threshold for data generated at each site was as follows: between 5,000 and 25,000 for SRDRI, 5,000 for MCW, between 25,000 and 35,000 for UQ and between 7,500 and 10,000 for ITN.

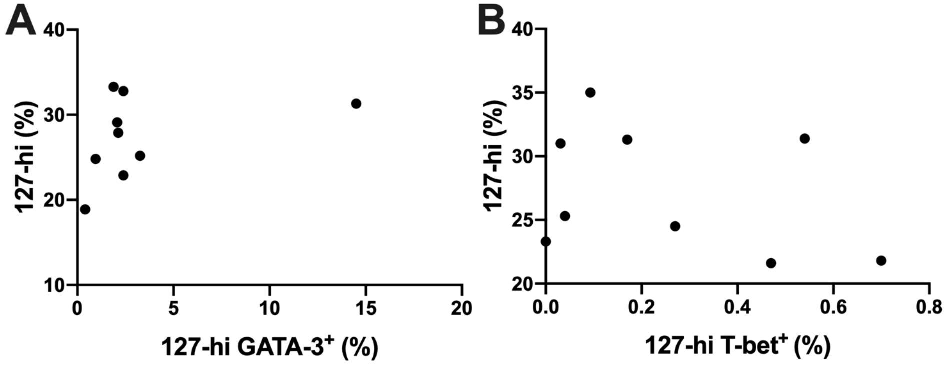

Supplemental Figure 2. The relative frequency of GATA-3+ 127-hi cells is not different in patients with different relative frequencies of 127-hi cells Data described in Figure 8 were re-analyzed to determine whether there is a correlation between the relative frequency of 127-hi cells in each patients and the frequency of those 127-hi cells that express either GATA-3 (A, n = 9), or T-bet (B, n = 9) or RORγt (C, n = 9).

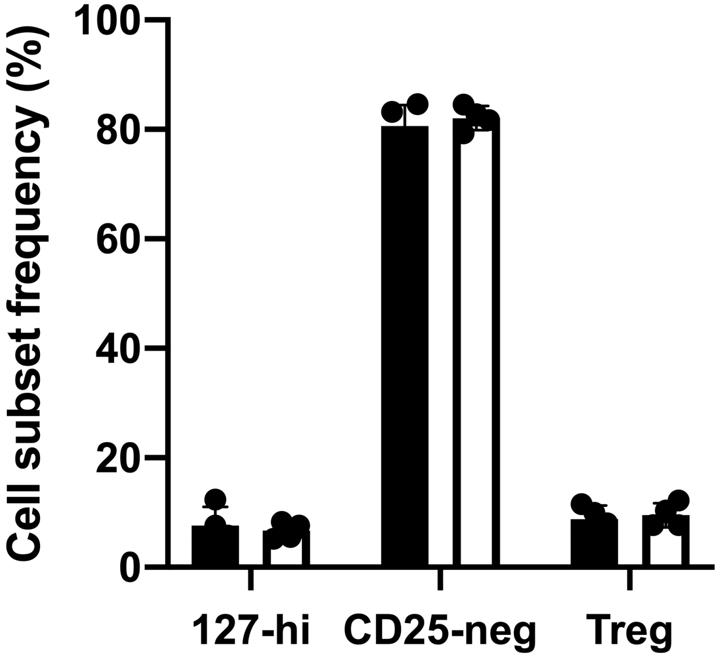

Supplemental Figure 3. The relative frequency of 127-hi, CD25-neg and Treg cells from thawed PBMC was not different from freshly isolated PBMC Whole blood was collected from healthy adult donors and PBMC were isolated (n = 4). Vials of PBMC were either frozen (open bars) immediately or labeled for CD3, CD4, CD25 and CD127 (closed bars). The relative frequency of 127-hi, CD25-neg and Treg cells were determined and compared to the relative frequency of the same cell subsets from the same donors, at least 2 weeks after freezing.

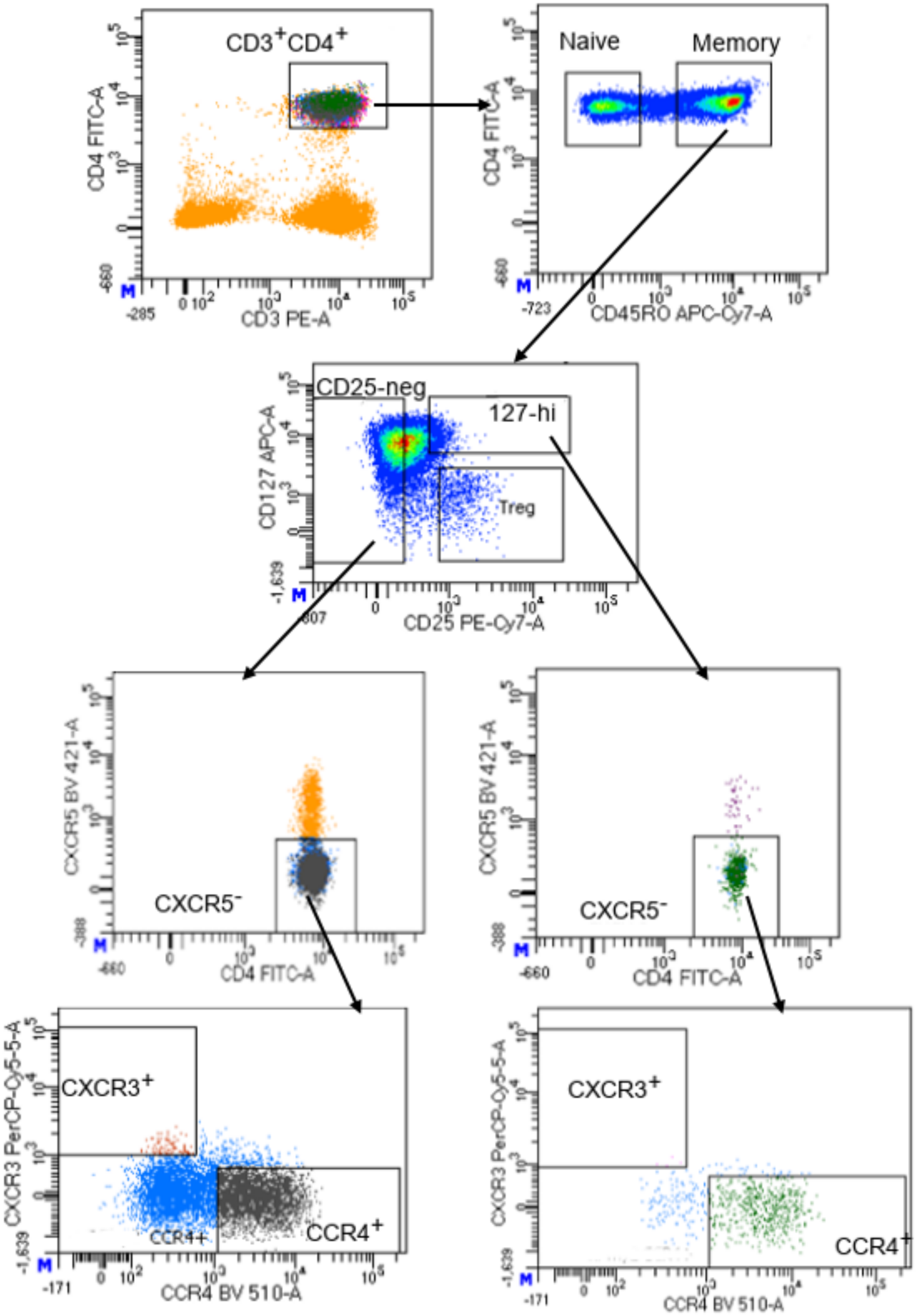

<u>Supplemental Figure 4.</u> <u>A schematic for sorting pre-Th1 and pre-Th2 127-hi and CD25-neg cells</u> PBMC are stained for CD3 CD4 CD45RO CXCR5 CXCR3 CCR4. The sort schematic for the isolation of 127-hi pre-Th2 and pre-Th1 and CD25-neg pre-Th2 and pre-Th1 is displayed.

Supplemental Table 1 denotes the total number of cells derived for each sort population from total PBMCs from people with T1D.

Cell Subset	Cell Count									
	T1D #1	T1D #2	T1D #3	T1D #4	T1D #5	T1D #6	T1D #7	T1D #8	T1D #9	T1D #10
CD25-neg preTh1	171367	29778	47659	12897	10627	20986	30757	30161	32636	79526
CD25-neg preTh2	64923	143914	94091	67518	530593	162263	197447	123891	98462	111158
127-hi preTh1	5489	330	511	131	93	544	676	620	672	1080
127-hi preTh2	5712	18203	34637	11443	36173	35156	32663	24269	5110	9756

Supplemental Table 2 lists all the antibodies used during this study

Antigen	*Flurochorme*	*Clone*	*Vendor*	*Catalog #*	*Antibody registry #*
**Panel run at Univ of Queensland; Collaborator: Dr. R. Thomas**					
Live/Dead	BV510	Not applicable	Invitrogen	L34957	Not applicable
CD3	FITC	OKT3	BioLegend	317306	AB_571907
CD4	AF700	OKT4	BioLegend	317426	AB_571943
CD25	PE	BC96	BioLegend	302606	AB_314276
CD127	BV421	A019D5	BioLegend	351310	AB_10960140
CD45RO	APC-Cy7	UCHL1	BioLegend	304228	AB_10895897
CD45RA	PerCP-Cy5.5	HI100	BioLegend	304122	AB_893357
CXCR3 (CD183)	APC	G025H7	BioLegend	353708	AB_10983064
CXCR5 (CD185)	BUV395	RF8B2	BD Biosciences	740266	AB_2740008
CCR4 (CD194)	BV605	L291H4	BioLegend	359418	AB_2562483
**Panel at ITN for the T1DAL study; Collaborator: Dr. E. Serti**					
CD3	AF700	UCHT1	BD Biosciences	557943	AB_396952
CD4	V500	RPA - T4	BD Biosciences	560769	AB_1937331
CD25	PE-CF594	M - A 251	BD Biosciences	562403	AB_11151919
CD127	BV421	A019D5	BioLegend	351310	AB_10960140
CD45RO	BV650	UCHL1	BioLegend	304231	AB_2561359
CD45RA	PE-Cy7	HI100	BioLegend	304126	AB_10708879
CCR7	PE	G043H7	BioLegend	353204	AB_10913813
CD2	PerCP-e710	RPA-2.10	eBiosciences	46-0029-42	AB_10670351
**Panel run at SDBRI; Samples provided by ITN**					
Human Treg Cocktail	FITC, PE-Cy7, AF647	SK3, 2A3, HIL-7R-M21	BD Biosciences	560249	AB_1645496
CCR7	PE	G043H7	BioLegend	353204	AB_10913813
CXCR3 (CD183)	PerCP- Cy5.5	1C6	BD Biosciences	560832	AB_2033945
CD45RO	APC-H7	UCHL1	BD Biosciences	561137	AB_10562194
CXCR5 (CD185)	BV421	RF8B2	BD Biosciences	562747	AB_2737766
CCR4 (CD194)	BV510	1G1	BD Biosciences	563066	AB_2737984
CD3	BV605	SK7	BD Biosciences	563217	AB_2714001
GATA3	PE	16E10A23	BioLegend	653804	AB_2562723
T-bet	BV421	4B10	BioLegend	644816	AB_10959653
RORγt	PE	AFKJS-9	eBiosciences	12-6988-82	AB_1834470
**Panel run at SRDRI; Collaborator: Dr. M. Battaglia**					
CD3	PerCP-Cy5.5	SK7	BD Biosciences	345766	AB_2783791
CD4	PE-Cy7	SK3	BD Biosciences	557852	AB_396897
CD25	APC	2A3	BD Biosciences	340907	AB_2819021
CD127	PE	R34.34	Beckman Coulter	IM1980U	AB_131301
CD45	Pacific Blue	HI30	BioLegend	304022	AB_493655
CD45RA	FITC	HI100	BD Biosciences	555488	AB_395879
**Panel run at MCW; Collaborator: Dr. M. Hessner**					
CD3	BUV395	UCHT1	BD Biosciences	563546	AB_2744387
CD4	APC-H7	RPA-T4	BD Biosciences	560158	AB_1645478
CD25	PE-Cy7	2A3	BD Biosciences	335789	AB_399968
CD127	PE	HIL-7R-M21	BD Biosciences	557938	AB_2296056
CD45RO	FITC	UCHL1	BD Biosciences	555492	AB_395883
CD45RA	BV510	HI100	BD Biosciences	563031	AB_2722499
Foxp3	APC	PCH101	eBiosciences	14-4776-82	AB_467554
Live/Dead	BV421	Not applicable	Invitrogen	L34955	Not applicable
